# Sorafenib inhibits interferon production by plasmacytoid dendritic cells in hepatocellular carcinoma

**DOI:** 10.1186/s12885-022-10356-2

**Published:** 2022-11-30

**Authors:** Xinning Zhang, Yong Xu, Guodong Zhao, Rong Liu, Haisheng Yu

**Affiliations:** 1 Faculty of Hepato-Pancreato-Biliary Surgery, Chinese PLA General Hospital, Beijing, China; 2 Institute of Hepatobiliary Surgery of Chinese PLA, Beijing, China; 3 Key Laboratory of Digital Hepetobiliary Surgery, Chinese PLA, Beijing, China; 4 Chinese PLA General Hospital and Chinese PLA Medical School, Beijing, China; 5 Guangzhou Eighth People’s Hospital, Guangzhou Medical University, Guangzhou, China

**Keywords:** Plasmacytoid dendritic cells, Hepatocellular carcinoma, Sorafenib, IFNα

## Abstract

**Background:**

Sorafenib is a multi-kinase inhibitor that shows antitumor activity in advanced hepatocellular carcinoma. Sorafenib exerts a regulatory effect on immune cells, including T cells, natural killer cells and dendritic cells. Studies have shown that plasmacytoid dendritic cells (pDCs) are functionally impaired in cancer tissues or produce low type I interferon alpha (IFNα) in cancer microenvironments. However, the effects of sorafenib on the function of pDCs have not been evaluated in detail.

**Methods:**

Normal and patient PBMCs were stimulated with CpG-A to evaluate IFNα production with Flow cytometry and ELISA.

**Result:**

We analyzed the production of IFNα by PBMCs in patients with advanced HCC under sorafenib treatment. We found that sorafenib-treated HCC patients produced less IFNα than untreated patients. Furthermore, we demonstrated that sorafenib suppressed the production of IFNα by PBMCs or pDCs from heathy donors in a concentration-dependent manner.

**Conclusion:**

Sorafenib suppressed pDCs function. Given that sorafenib is a currently recommended targeted therapeutic agent against cancer, our results suggest that its immunosuppressive effect on pDCs should be considered during treatment.

## Background

Hepatocellular carcinoma (HCC) is one of the most common malignant tumors worldwide. Surgical resection and liver transplantation are the main curative treatment modalities for HCC. However, in most cases, curative treatment is not feasible because patients are diagnosed with HCC at an advanced stage; consequently, the overall effect of treatment against HCC at this stage is poor [1]. Although nonsurgical treatments for HCC (e.g., radiofrequency ablation and transcatheter arterial chemoembolization) are also available, the overall survival rate of patients is not satisfactory [2]. Sorafenib is thought to inhibit tumor growth via anti-angiogenesis, cell cycle arrest, and apoptosis [3]. In two pivotal phase III trials, sorafenib significantly improved overall survival in patients with advanced HCC [4, 5]. Based on these results, sorafenib is recommended as the standard first-line systemic therapy for patients with advanced HCC.

Sorafenib is currently the systemic agent approved for the treatment of HCC [6]. In addition to its anticancer activity, sorafenib also exerts a regulatory effect on immune cells, including T cells [7], natural killer (NK) cells [8], macrophages [9], and dendritic cells (DCs) [10]. It has been reported that sorafenib modulate the immunobiological activity of immune cells in HCC. Pharmacological doses of sorafenib specifically enhance the activation of CD4^+^CD25^−^ effector T cells and blocked the function in patients with HCC [11]. Sorafenib also could suppress progression of HCC by reducing the number of myeloid-derived suppressor cells and NK cells in mice [12, 13]. It can also inhibit the functions of DCs through inhibiting secretion of cytokines [10], or alter the differentiation of human monocytes to DCs through VEGF [14].

As a major subset of DCs, plasmacytoid dendritic cells (pDCs) express high levels toll-like receptor 7 (TLR7) and TLR9. Accordingly, they produce large amounts of type I interferon (IFN-I) upon stimulation with nucleic-acid ligands of TLR7/TRL9, as well as the upregulation of costimulatory molecules [15]. PDCs have direct tumoricidal activity though a granzyme B- and tumor necrosis factor-related apoptosis, and indirect activity through the activation of NK cells [16]. Studies have shown that pDCs are clinically important in patients, for example, pDCs are functionally impaired in cancer tissues or sentinel lymph nodes, or produce low IFNα in cancer microenvironments [17]. The numbers of pDCs are increased in the tumor tissues and decreased in the blood of patients with HCC [18, 19]. These cells cooperatively establish the immunosuppressive environment to promote the progression of liver cancer by increasing the number of regulatory T cells [20]. Therefore, liver pDCs exhibit both immune-augmenting and immunosuppressive functions depending on the environmental conditions.

However, thus far, the effects of sorafenib on the function of pDCs have not been evaluated in detail. In this study, we found that treatment with sorafenib inhibited the ex vivo production of IFNα by pDCs in patients with HCC. Furthermore, sorafenib can inhibit the production of IFNα by CpG-A stimulation in vitro by PBMCs or purified pDCs.

## Methods

### Patient information

Ethics Committee approval was obtained from the Institutional Ethics Committee of Chinese PLA General Hospital to the commencement of the study (Ethics approval: S2016–098-01), and participants were enrolled after they provided informed consent. A total of 42 patients with advanced HCC (Barcelona Clinic Liver Cancer [BCLC] C stage) who received treatment at the Chinese People’s Liberation Army General Hospital (Beijing, China) between September 2017 and May 2018 were enrolled in this study. All patients were diagnosed through histological and cytological analyses, or based on persistently elevated serum levels of alpha-fetoprotein (≥400 ng/mL) with typical imaging findings. Other inclusion criteria for this study included unresectable recurrent HCC according to the BCLC staging classification and Child–Pugh class A with Eastern Cooperative Oncology Group-Performance Status (ECOG-PS) of 0. The protocol of this study was approved by the Chinese People’s Liberation Army General Hospital, and all patients provided written informed consent. There are seven blood samples in the study group were tested for IFNα production by ELISA, nine blood samples in the study group were tested for pDCs number by FACS, eight blood samples in the study group were tested for IFNα production by intracellular staining. Blood samples from the control group were taken at the same time in different experiments.

### PBMCs from blood

All blood samples were obtained from Chinese PLA General Hospital. Peripheral blood mononuclear cells (PBMCs) were isolated immediately from fresh blood by Ficoll gradient centrifugation, according to standard density-gradient centrifugation methods (GE Healthcare).

### PDCs purification

PBMCs were stained with a mixture of biotin anti-CD3, anti-CD16 and anti-CD19. The lineage negative cells (CD3^−^CD16^−^CD19^−^PBMCs) were enriched by Streptavidin MicroBeads (Miltenyi Biotech). The enriched cells were stained APC-Cy7 anti-HLA-DR and PE anti-CD123, and HLA-DR^+^CD123^+^ pDCs were sorted using BD FACSAria III (BD Biosciences). The purity of sorted cells was rechecked by FACS again on a BD FACSAria III.

### Reagents and cell culture

Sorafenib tosylate was purchased from Bayer Healthcare (Leverkusen, Germany). The agent was dissolved in dimethyl sulfoxide (DMSO) and added to the culture media in concentrations ranging from 1 to 3 μg/mL, which corresponds to the serum levels achieved in treated patients [21, 22]. In each case, equal amounts of DMSO were added as a control to exclude the effects induced by the solvent. PBMCs or purified pDCs were cultured in RPMI 1640 (Hyclone) containing 10% fetal calf serum at 2 × 10^5^ PBMCs or 1 × 10^4^ pDCs per 200 uL in round-bottomed 96-well culture plates. Subsequently, these cells were pretreated with sorafenib or DMSO (control) for 2 h prior to stimulation with 2.5 uM CpG-A (Invivogen). After 6 or 24 h of culture, cells and supernatants were harvested for further analysis.

### Enzyme-linked immunosorbent assay (ELISA)

Cells were incubated in a 96-well plate alone or with different concentrations of sorafenib, and cell culture supernatants were collected and frozen at − 80 °C for later use. Measurement of IFNα levels was performed using the human cytokine detection system (Millipore) according to the instructions provided by the manufacturer.

### Intracellular cytokine analysis

PBMCs were pretreated with sorafenib or DMSO for 2 h prior to stimulation with 1 μM CpG-A (Invivogen) for 6 h in the presence of 10 mg/ml brefeldin A (BioLegend) for the final 3 h. Then, the cytokines IFNα were detected via intracellular staining [23], and examined using the LSRFortessa (BD Biosciences). The data were analyzed through the Summit software (Dako Cytomation).

### Statistical analysis

The GraphPad Prism software (Version 9.0) was used for statistical analysis. Data are shown as the mean ± S.D. of independent experiments, and each experiment was repeated at least thrice. Statistically significant differences were determined by unpaired two-tailed t tests. A *p*-value < 0.05 denoted statistical significance.

## Results

### Characteristics of patients and disease

Fourteen patients received two tablets of sorafenib (200 mg tablet) twice daily for at least 3 months; blood samples were collected from these patients. As control, 28 patients with HCC not treated with sorafenib, were also included. The response to treatment with sorafenib was evaluated using PBMCs obtained from the study groups. None of the patients received anticancer or antiviral therapy at the time of blood sample collection. The characteristics of patients and their diseases (age, sex, BCLC stage, Child–Pugh classification, recurrence of HCC, prior history of hepatitis) are listed in Table 1.

**Table 1:** The basic demographic and disease characteristics

	HCC + Sroafenib *n =* 14	HCC *n =* 28	P
Median age (range)	57 years (36–74 years)	51 years (38–74 years)	> 0.05
gender			
Male	14	23	> 0.05
Female	–	5	–
HBV infection	6	12	> 0.05
HCV infection	–		–
Recurrence HCC	14	28	> 0.05
Prior anticancer treatment			
Yes	14	28	> 0.05
No	–	–	–
BCLC stage			
A	–	–	–
B	–	–	–
C	14	28	> 0.05
Child-Pugh			
A	14	28	> 0.05
B	–	–	–
C			
Ascites	no	no	–
White blood cell count	4.6 × 10^9^	5.6 × 10^9^	> 0.05

The characteristics of patients and their diseases including age, gender, prior history of hepatitis, prior history anticancer treatment, BCLC stage, Child-Pugh classification. There is no significant difference in any of the characteristics between sorafenib treated and non-treated groups

### Sorafenib inhibited IFNα production of PBMCs from HCC patients

**Fig. 1:**
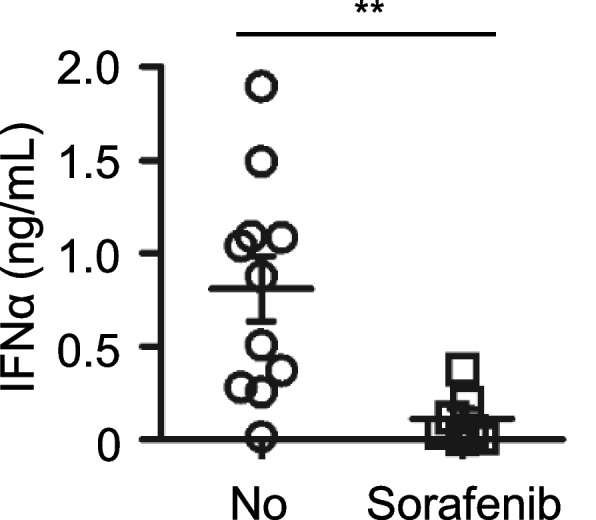
Sorafenib inhibits IFNα production by PBMCs in patients with HCC. PBMCs obtained from patients with advanced HCC were stimulated with CpG-A for 24 h. Seven patients received two tablets of sorafenib (200 mg tablet) twice daily for at least 3 months; 11 patients with advanced HCC were included as control. IFNα production was measured by ELISA. Each dot represents an individual donor, and the bars represent the mean ± S.D. **p < 0.01

**Fig. 2:**
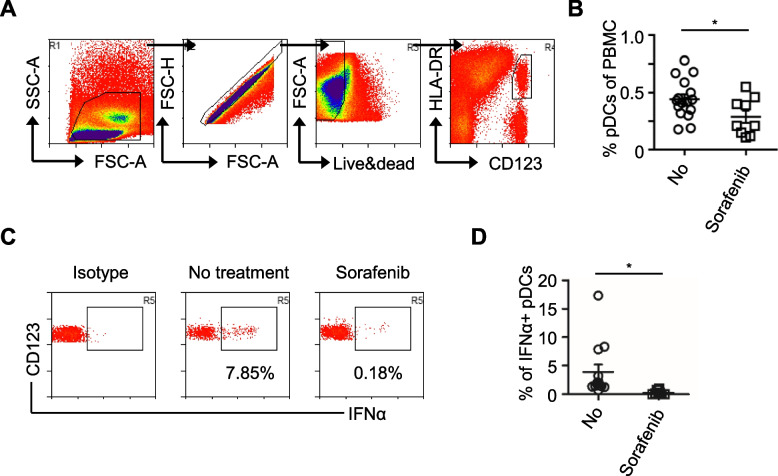
Decreased number and less IFNα of PBMCs from HCC patients treated with sorafenib. **A** Gating strategy for human pDCs in peripheral blood. Cells were first gated by FSC-A and SSC-A and doublets were excluded by the FSC-H and FSC-A. Exclusion of dead cells used live/dead staining. The pDCs were defined as HLA-DR^+^ CD123^+^ cells. **B** Summarized data (A) indicating the percentage of pDCs. Nine donors treated with sorafenib and 16 untreated donors were analyzed in six experiments. Each dot represents an individual donor, and the bars represent the mean ± S.D. *p < 0.05. **C** PBMCs collected from patients treated or not treated with sorafenib were stimulated with CpG-A for 6 h. Intracellular staining was used to analyze the production of IFNα. **D** Summarized data (C) indicating the production of IFNα. Eight donors treated with sorafenib and 13 untreated were analyzed in six experiments. Each dot represents an individual donor, and the bars represent the mean ± S.D. *p < 0.05

Sorafenib affects the quantity and quality of immune cells involved in antitumor immunity [10, 17]. To assess the impact of sorafenib, PBMCs obtained from patients with advanced HCC who received or did not receive sorafenib were stimulated with CpG-A. The results revealed that sorafenib reduced the secretion of IFNα by PBMCs collected from patients with HCC versus those obtained from untreated patients with HCC (Fig. 1). High production of IFNα upon stimulation with TLR7 or TLR9 ligands is the key characteristic of pDCs. It is essential to investigate the reason of IFNα reduction whether sorafenib affects the function of pDCs in patients with HCC. To investigate this hypothesis, we first determined the percentage of pDCs in PBMCs obtained from patients treated or not treated with sorafenib. The gating strategy for pDCs is shown in Fig. 2A. The results revealed that the percentage of pDCs in PBMCs decreased in sorafenib-treated HCC patients compared with those in the untreated group (Fig. 2B). These findings suggest that sorafenib reduces the number of pDCs in patients with HCC, potentially contributing to the inhibition of IFNα production.

To further verify whether sorafenib directly inhibited the production of IFNα by pDCs, we detected the levels of IFNα in patients with HCC treated or not treated with sorafenib. PBMCs were stimulated with CpG-A and IFNα production by pDCs was analyzed by intracellular staining. The data revealed that the percentage of IFNα-producing pDCs was significantly reduced in HCC patients treated with sorafenib (Figs. 2C, D). Taken together, these data indicated that low IFNα production of HCC patients may be caused by decreasing the percentage of pDCs and inhibiting IFNα production by sorafenib.

### Sorafenib inhibited the production of IFNα in PBMCs from heathy donors

**Fig. 3:**
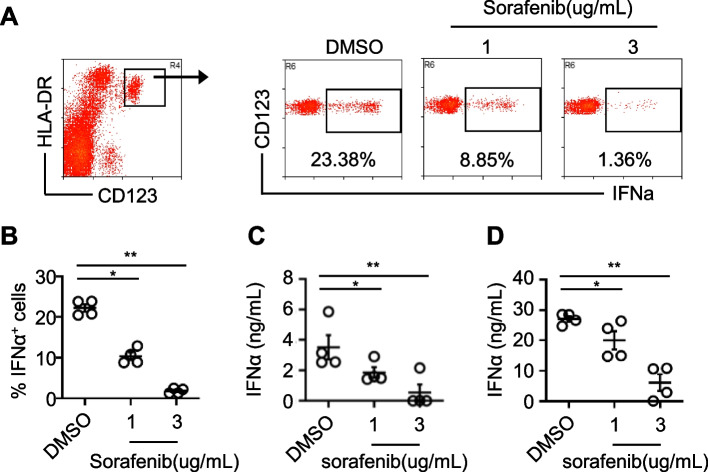
Sorafenib inhibits the CpG-A-induced production of IFNα by PBMCs or pDCs from healthy donor. **A** Healthy fresh PBMCs (2 × 10^6^ cells per well) seeded in 48-well plates were pretreated with the indicated concentrations of sorafenib (1 and 3 μg/mL) or DMSO (control) for 2 h prior to the stimulation with CpG-A (2.5 μM) for 6 h. BFA was added for the last 3 h of the culture. The amount of intracellular IFNα was determined by flow cytometry. The results are representative of two independent experiments. **B** The combined data (A) from two independent experiments involving four donors are shown. Each dot represents an individual donor, and the bars represent the mean ± S.D. *p < 0.05, **p < 0.01. ***C**** ELISA* showing that sorafenib inhibits the production of IFNα by PBMCs. PBMCs (2 × 10^5^ cells per well) seeded in 96-well plates were pretreated with sorafenib at indicated doses for 2 h prior to stimulation with CpG-A for 20 h. The levels of IFNα in the culture supernatant were quantified using ELISA. Data obtained from four donors were summarized. The bars represent the mean ± S.D. *p < 0.05, **p < 0.01. **D** PDCs were sorted using FACS Aria III. The purified pDCs were pretreated with sorafenib for 2 h at indicated concentrations prior to stimulation with CpG-A for 20 h. The levels of IFNα in the culture supernatant were quantified using ELISA. Data from four donors were summarized. The bars represent the mean ± S.D. *p < 0.05, **p < 0.01

To investigate whether sorafenib inhibited healthy human pDCs, we first examined the effects of it on the secretion of IFNα in healthy PBMCs. Heathy PBMCs were stimulated with CpG-A for 6 h before pretreatment with sorafenib at two concentrations (1 and 3 μg/mL) for 2 h. Subsequently, IFNα production was analyzed by flow cytometry and the gating strategy is shown in Fig. 3A. We found that sorafenib at pharmacologic concentrations inhibited the production of IFNα by healthy PBMCs in a dose-dependent manner (Fig. 3A&B). Next, we also examined the levels of IFNα in the supernatant of PBMCs stimulated with CpG-A using ELISA (Fig. 3C). The results showed that sorafenib can significantly inhibit the production of IFNα in healthy PBMCs. To eliminate the possibility of the interaction of non-pDCs cells, we isolated pDCs from PBMCs and incubated with sorafenib at varying concentrations (1 and 3 μg/mL) for 2 h prior to stimulation with CpG-A for 20 h. The supernatant was collected and the levels of IFNα were detected by ELISA. We found that sorafenib significantly inhibited the CpG-A-induced production of IFNα in purified pDCs in a dose-dependent manner (Fig. 3D). These findings indicated that sorafenib can directly inhibit IFNα production.

### Sorafenib does not affect the viability of pDCs

**Fig. 4:**
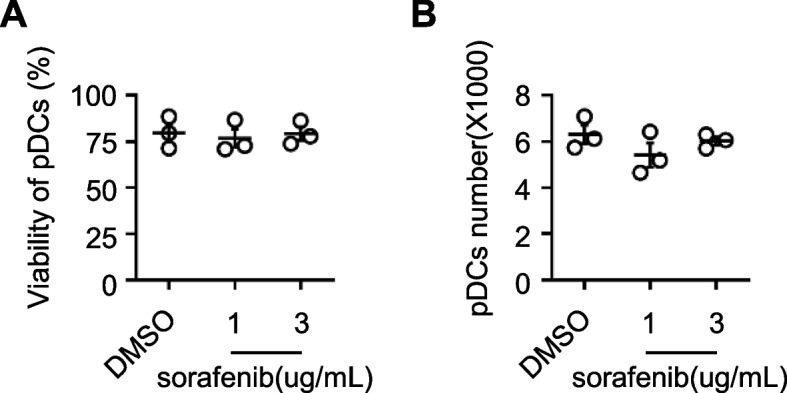
Sorafenib does not promote pDCs death. **A** PDCs (3 × 10^4^) were pretreated with sorafenib (1 and 3 μg/mL) or DMSO (control) for 2 h, prior to stimulation with CpG-A (2.5 μM) for 24 h. The viability (**A**) and number (**B**) of pDCs were determined through a Guava Via count (Luminex). The pDCs from Fig. 3.D in these experiments, and the bars represent the mean ± S.D

Sorted pDCs were pretreated with sorafenib or DMSO prior to stimulation with by CpG-A. The viability and number of pDCs were determined using the Guava Viacount (Luminex). The results show sorafenib does not decreased the viability and number of pDCs (Figs. 4A&B). Collectively, these data indicated that sorafenib inhibits function of pDCs at pharmaco-physiological concentrations (1–3 μg/mL).

## Discussion

The multi-kinase inhibitor sorafenib is the first systemic drug approved for the treatment of advanced HCC. It has been shown that treatment with this agent confers a survival benefit to patients of approximately 3 months [4]. Currently, sorafenib remains the standard first-line systemic therapy for patients with advanced HCC [6]. Meanwhile, sorafenib affects the quantity and quality of immune cells involved in antitumor immunity [7–10, 14]. In this study, we found that sorafenib inhibited the production of IFNα by pDCs in patients with HCC. Furthermore, this impairment was observed in PBMCs and purified pDCs stimulated with CpG-A in vitro.

PDCs are generally accepted as the major type I IFN-producing cells of the immune system. These type I IFNs promote the functions of other immune cells and link innate and adaptive immune responses, including antitumor T cells responses [15]. Consequently, they can initiate protective immunity through maturation of DCs and subsequent activation of T cells and NK cells [24]. Current research suggests that pDCs have the capacity to induce apoptosis of neoplastic cells; therefore, they can contribute to cancer surveillance [17]. However, pDCs in cancer tissues have limited ability to produce IFNα and play minor immunosuppressive roles in tumor immunity [25–28]. Interestingly, tolerogenic pDCs may even contribute to tumor progression. Our results suggest that the reduced production of IFNα by pDCs after treatment with sorafenib may have adverse effects on the immune surveillance of tumors.

HCC is the most common type of liver cancer. Previous reports indicated that the percentage of pDCs is increased in the tumor tissues and decreased in the blood of patients with HCC [17, 18]. PDCs in patients with HCC display numerical deficiencies and an immature phenotype, while tolerogenic pDCs are increased in patients with advanced HCC. These pDCs may establish the immunosuppressive environment to promote the progression of liver cancer [19]. An intertumoral infiltration of pDCs is predictive of poor outcome for patients undergoing curative resection for HCC. This is possibly due to the induction of an immune tolerogenic and inflammatory tumor microenvironment [29]. Thus, sorafenib has exhibited immunosuppressive effects on pDCs, which may contribute to the development of a tumor immunosuppressive microenvironment in patients with HCC.

## Conclusions

This study indicated that sorafenib inhibits the production of IFNα by pDCs. The present findings further enhance our understanding of the safety profile of sorafenib and the immunosuppressive effect of sorafenib should be considered during the treatment of cancer.

## Data Availability

The datasets used and/or analyzed during the current study available from the corresponding author on reasonable request.

## Abbreviations

HCC: Hepatocellular carcinoma
IFNα: Interferon-α
pDCs: Plasmacytoid Dendritic Cells
